# Auto-thiophosphorylation activity of Src tyrosine kinase

**DOI:** 10.1186/s12858-016-0071-z

**Published:** 2016-07-07

**Authors:** M. Zulema Cabail, Emily I. Chen, Antonius Koller, W. Todd Miller

**Affiliations:** Department of Physiology and Biophysics, School of Medicine, Stony Brook University, Stony Brook, NY 11794 USA; Biological Sciences Department, SUNY College at Old Westbury, Old Westbury, NY 11568 USA; Proteomics Shared Resource at the Herbert Irving Comprehensive Cancer Center, New York, NY 10032 USA; Department of Pharmacology, Columbia University Medical Center, New York, NY 10032 USA

**Keywords:** Tyrosine kinase, Src, Thiophosphate, Autophosphorylation, Phosphatase

## Abstract

**Background:**

Intermolecular autophosphorylation at Tyr416 is a conserved mechanism of activation among the members of the Src family of nonreceptor tyrosine kinases. Like several other tyrosine kinases, Src can catalyze the thiophosphorylation of peptide and protein substrates using ATPγS as a thiophosphodonor, although the efficiency of the reaction is low.

**Results:**

Here, we have characterized the ability of Src to auto-thiophosphorylate. Auto-thiophosphorylation of Src at Tyr416 in the activation loop proceeds efficiently in the presence of Ni^2+^, resulting in kinase activation. Other tyrosine kinases (Ack1, Hck, and IGF1 receptor) also auto-thiophosphorylate in the presence of Ni^2+^. Tyr416-thiophosphorylated Src is resistant to dephosphorylation by PTP1B phosphatase.

**Conclusions:**

Src and other tyrosine kinases catalyze auto-thiophosphorylation in the presence of Ni^2+^. Thiophosphorylation of Src occurs at Tyr416 in the activation loop, and results in enhanced kinase activity. Tyr416-thiophosphorylated Src could serve as a stable, persistently-activated mimic of Src.

**Electronic supplementary material:**

The online version of this article (doi:10.1186/s12858-016-0071-z) contains supplementary material, which is available to authorized users.

## Background

Autophosphorylation is a common mechanism by which the activities of eukaryotic protein kinases are controlled [1, 2]. The canonical protein kinase fold consists of two lobes separated by a deep cleft into which ATP binds [3]. Protein and peptide substrates bind in an extended conformation at the entrance to this cleft. A flexible protein segment between the lobes called the “activation loop” interacts with protein and peptide substrates. In many serine/threonine kinases (e.g., PKA), and tyrosine kinases (e.g., Src), this loop contains one or more phosphorylation sites. Autophosphorylation within the activation loop stabilizes a conformation that allows substrate binding, and promotes kinase activity [1–3]. In principle, autophosphorylation can be either an intermolecular reaction between two kinase molecules (also called autophosphorylation “in trans”) or an intramolecular reaction within one kinase (“cis”). For tyrosine kinases where this has been examined explicitly, autophosphorylation is intermolecular [4–6]. This mode of kinase regulation appears to be evolutionarily ancient, as (for example) Src kinases from unicellular choanoflagellates and filastereans are activated by autophosphorylation at the position corresponding to Tyr416 of c-Src [7, 8] (chicken Src numbering is used throughout this paper). The number and positioning of phosphate residues within the activation loop varies from kinase to kinase.

Several serine/threonine and tyrosine protein kinases have the ability to catalyze thiophosphorylation of peptide and protein substrates (by the use of ATPγS rather than ATP as cosubstrate) [9–12]. Thiophosphorylated proteins are more metabolically stable than their phosphorylated counterparts; in particular, they are resistant to cellular phosphatases [13, 14]. This has facilitated their use in proteomic investigations of kinase activity, where some phosphoproteins are intrinsically unstable, or are modified at low stoichiometry [15]. The thiophosphoryl group can also be further functionalized for proteomic studies [9], or for specific “caging” reactions to produce molecules that are released upon photolysis [16].

Although a number of protein kinases have the capacity to use ATPγS as a phosphodonor, the kinetic efficiency of protein kinase reactions with ATPγS is typically much lower than that for comparable reactions with ATP. This is particularly true for tyrosine kinases. To circumvent this problem, we and others have shown that tyrosine kinases can catalyze thiophosphorylation of peptide substrates in the presence of divalent transition metals (e.g., Co^2+^ or Ni^2+^) in the reaction buffer rather than magnesium [10, 16]. This is thought to be due to the increased relative affinity of Co^2+^ or Ni^2+^ toward sulfur in nucleotide complexes, as compared with Mg^2+^, which has a strong preference for binding oxygen over sulfur [17, 18]. Thus, for Csk tyrosine kinase, k_cat_ for substrate phosphorylation was comparable for ATP vs. ATPγS in the presence of thiophilic divalent metals, but k_cat_ for ATPγS was greatly reduced in the presence of magnesium or manganese. This was attributed to the important role of γ-phosphoryl bonding and salt bridging in the Csk reaction transition state [10]. Similarly, we showed that an SH2-binding peptide could be thiophosphorylated by Hck kinase in the presence of cobalt [16].

Previous work in this area has focused primarily on the kinase-catalyzed thiophosphorylation of peptide substrates. Gel-based methods have been used to demonstrate incorporation of thiophosphate into kinase substrates and kinases, including Src and Abl [19, 20]. Here, we have characterized the ability of Src to catalyze intermolecular auto-thiophosphorylation. We report that auto-thiophosphorylation of Src at Tyr416 in the activation loop proceeds efficiently in the presence of Ni^2+^, stabilizing the active conformation and resulting in kinase activation. Tyr416-thiophosphorylated Src is resistant to dephosphorylation by PTP1B phosphatase, and could serve as a stable, persistently-activated mimic of Src.

## Results

**Fig. 1:**
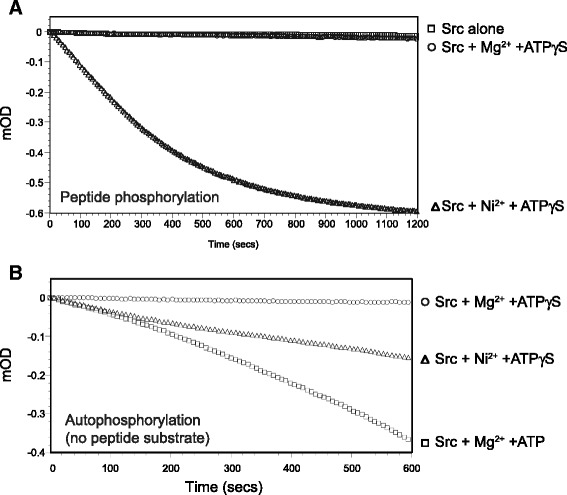
Thiophosphorylation and auto-thiophosphorylation catalyzed by Src kinase. **a**. The activity of Src kinase domain (450 nM) toward a synthetic peptide substrate (AEEEIYGEFEAKKKKG, 800 μM) was measured using the continuous spectrophotometric assay. The reactions contained no ion/nucleotide (*squares*), 10 mM MgCl_2_ and 2 mM ATPγS (*circles*), or 10 mM NiCl_2_ and 2 mM ATPγS (*triangles*). Absorbance data were recorded every 8 s. **b**. The activity of Src (2.2 μM) was measured in the absence of peptide substrate using the continuous spectrophotometric assay. The reactions contained 10 mM divalent cation (Mg^2+^ or Ni^2+^) and 2 mM nucleotide (ATP or ATPγS), as indicated

In our previous studies [16], we showed that the Src family kinase Hck can catalyze thiophosphorylation of a peptide substrate in the presence of CoCl_2_. In those studies, an endpoint assay was used; we analyzed aliquots from reactions by analytical HPLC, and confirmed peptide thiophosphorylation by mass spectrometry. Here, we tested whether a continuous assay could be used to monitor peptide thiophosphorylation. Using a coupled spectrophotometric assay that measures NADH consumption, we found that Src catalyzed peptide thiophosphorylation in the presence of ATPγS and Ni^2+^, but not Mg^2+^ (Fig. 1a). Co^2+^ was much less efficient in these experiments (data not shown). We did not observe any consumption of NADH in the absence of Src, or in Src reactions without divalent cations (Fig. 1a).

Next, we wished to determine whether Src kinase could catalyze intermolecular *auto*-thiophosphorylation (as opposed to phosphorylation of an exogenous peptide substrate). The Src kinase used for these experiments was purified from bacteria, and contains very low levels of phosphorylation [21]. Using the continuous assay, we detected auto-thiophosphorylation activity in the presence of Ni^2+^ (Fig. 1b). The initial rate of auto-thiophosphorylation (0–100 s) was similar to that observed for Src autophosphorylation in the presence of ATP and Mg^2+^ (Fig. 1b); after 600 s, the overall rate of the NiATPγS reaction was roughly one-half that of the MgATP reaction. We observed minimal activity using ATPγS as cosubstrate with Mg^2+^ as the divalent cation. This is consistent with earlier studies in which MgATPγS did not support autoactivation of Src, and acted as a competitive inhibitor versus MgATP with K_i_ = 23 μM [6].

**Fig. 2:**
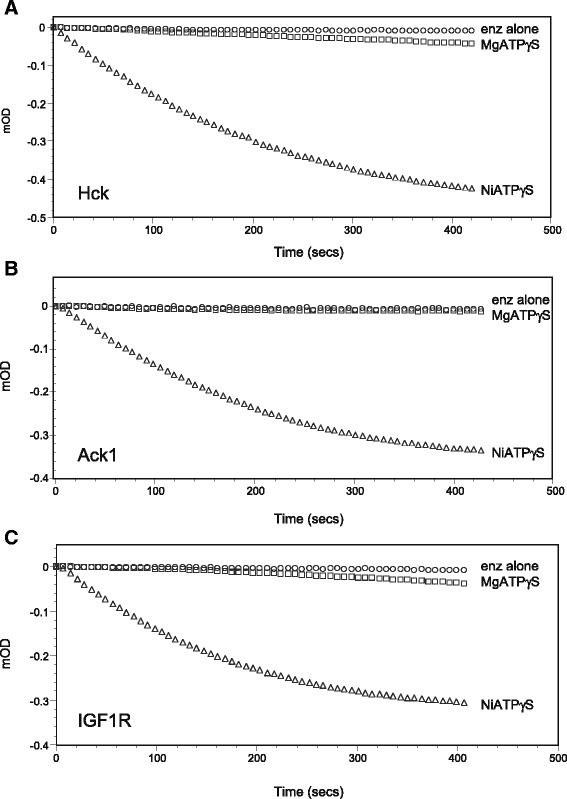
Auto-thiophosphorylation activity of other tyrosine kinases. Conditions for the continuous spectrophotometric assay were similar to Fig. 1. The reactions contained enzyme alone (*circles*), enzyme with 2 mM ATPγS and 10 mM MgCl_2_ (*squares*) or enzyme with 2 mM ATPγS and 10 mM NiCl_2_ (*triangles*). **a** Hck kinase (0.6 μM). **b** Ack1 (kinase-SH3 construct), 1.0 μM. **c** IGF1R kinase domain, 1.4 μM

We tested whether other tyrosine kinases can catalyze auto-thiophosphorylation. First, we examined Hck (hematopoetic cell kinase), another Src family kinase. Hck had robust auto-thiophosphorylation activity in the presence of Ni^2+^, but only minimal activity in the presence of Mg^2+^ (Fig. 2a). We obtained similar results for Ack1 (activated Cdc42-associated tyrosine kinase 1), a nonreceptor tyrosine kinase from a different family (Fig. 2b). The catalytic domain of the human insulin-like growth factor I receptor (IGF1R) also displayed auto-thiophosphorylation activity in the presence of NiATPγS, but only weak activity with MgATPγS (Fig. 2c).

**Fig. 3:**
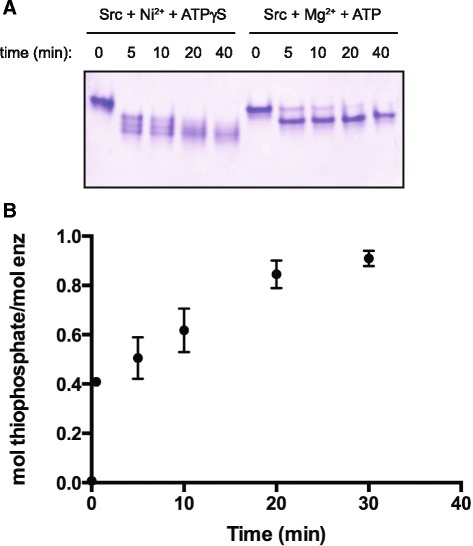
Measurements of the extent of Src auto-thiophosphorylation. **a** Src kinase domain was incubated in the presence of NiATPγS or MgATP, and the time course of reaction was monitored by nondenaturing gel electrophoresis. Detection was with Coomassie blue staining. **b** The stoichiometry of Src auto-thiophosphorylation was determined by carrying out reactions in the presence of [^35^S]-labeled ATPγS. Error bars show standard deviations. The experiments in panels **a** and **b** were carried out at least three times with similar results

As an initial measure of the extent of Src auto-thiophosphorylation, we carried out native gel electrophoresis. In the presence of Mg^2+^ and a high concentration of ATP (5 mM), we observed a nearly complete shift in the electrophoretic mobility of Src kinase after 20 min (Fig. 3a, right-hand lanes). This increased mobility is consistent with the behavior of Src and other tyrosine kinases after activation loop phosphorylation. Src also showed a pronounced shift in mobility upon incubation with Ni^2+^ and 5 mM ATPγS, although the pattern of bands was more complex in the initial phase of the reaction (Fig. 3a, left-hand lanes). Very little unphosphorylated Src was present after the reaction, suggesting that thiophosphorylation occurred with high stoichiometry. To confirm these findings, we carried out in vitro thiophosphorylation experiments with [^35^S]-labeled ATPγS. Src kinase incorporated 0.91 ± 0.03 mol of thiophosphate per mole of enzyme after 30 min of reaction with Ni^2+^ and [^35^S]-ATPγS (Fig. 3b). Even at the earliest time point we measured (1 min), Src had incorporated 0.4 mol thiophosphate/mol protein (Fig. 3b).

**Fig. 4:**
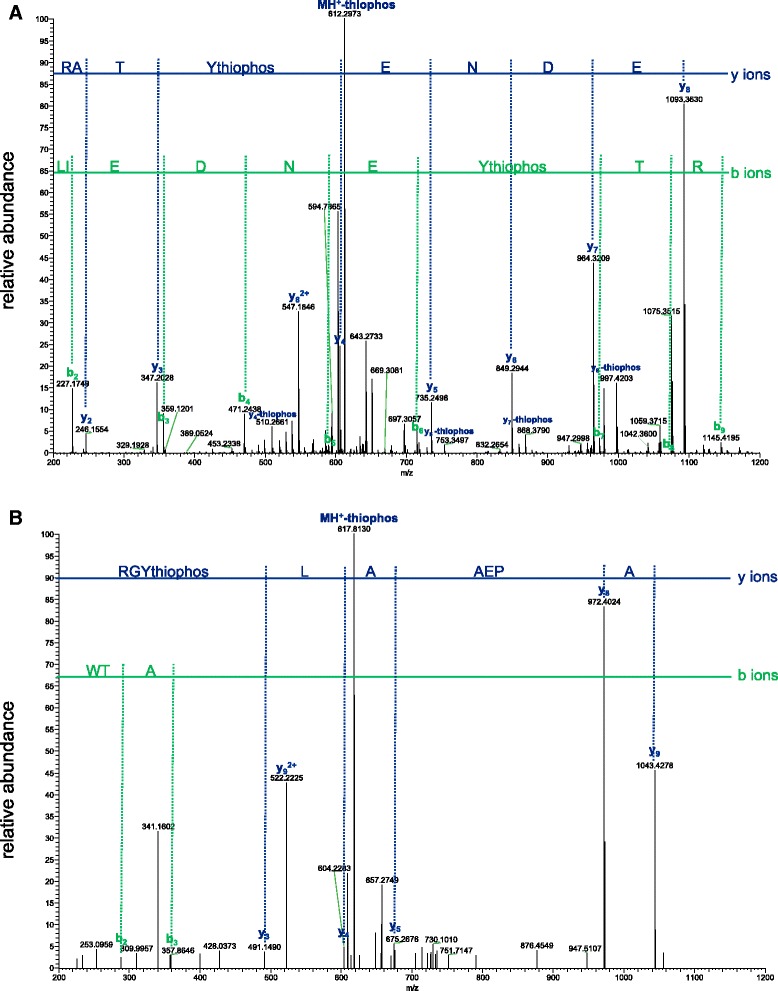
Mass spectrometric analysis of the sites of Src auto-thiophosphorylation. A sample of auto-thiophosphorylated Src was analyzed by SDS-PAGE. The gel band was excised, digested with trypsin, and analyzed by LC-MS/MS as described in Materials and Methods. **a** MS/MS scan of the peptide containing the major autophosphorylation site (Tyr416), LIEDNEYTAR. **b** MS/MS scan of the peptide containing the secondary thiophosphorylation site, WTAPEAALYGR

We determined the site of Src kinase auto-thiophosphorylation by tandem mass spectrometry. The major site of thiophosphorylation was in the sequence Leu-Ile-Glu-Asp-Asn-Glu-(thio)pTyr^416^-Thr-Ala-Arg, corresponding to the known site of autophosphorylation within the activation loop (Fig. 4a and Additional file 1: Figure S1). A secondary site of thiophosphorylation was found: Trp-Thr-Ala-Pro-Glu-Ala-Ala-Leu-(thio)pTyr^436^-Gly-Arg (Fig. 4b). By analyzing the areas under the curves of the chromatography elution profiles, we determined that 31 % of the signal for the Tyr416-containing peptide was the thiophosphorylated species, while only 0.06 % of the Tyr436-containing peptide was the thiophosphorylated species (Additional file 2: Figure S2). These data show that the replacement of ATP with ATPγS did not change Src’s preference for autophosphorylation within the activation loop.

**Fig. 5:**
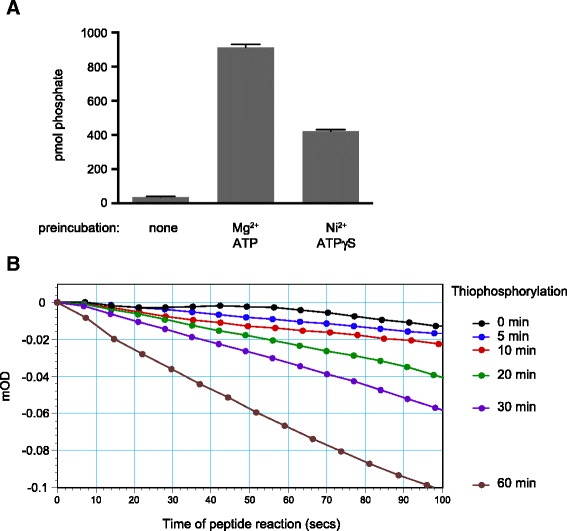
Activation of Src by auto-thiophosphorylation. **a** Unphosphorylated Src catalytic domain (2.2 μM) was preincubated for 15 min at 30 °C either alone, in the presence of 2 mM ATP and 10 mM MgCl_2_, or in the presence of 2 mM ATPγS and 10 mM NiCl_2_. Src activity was measured using the phosphocellulose paper assay in the presence of peptide (0.8 mM) and [^32^P]-ATP (0.4 mM). Error bars show standard deviations. **b** Unphosphorylated Src (1 μM) was preincubated for various lengths of time at 30 °C with 2 mM ATPγS and 10 mM NiCl_2_. Src activity was measured toward the synthetic peptide substrate (0.87 mM) using the continuous spectrophotometric assay

All Src family nonreceptor tyrosine kinases have a single tyrosine residue in the activation loop at the position corresponding to Tyr416 of Src. Intermolecular autophosphorylation at the conserved tyrosine triggers a large increase in enzyme activity [4, 6]. We compared the activities of bacterially-expressed, purified Src catalytic domain with samples that had been autophosphorylated (with Mg^2+^ and ATP) or auto-thiophosphorylated (with Ni^2+^ and ATPγS). After 15 min of reaction, we measured Src activity using a synthetic peptide substrate and [γ-^32^P]-ATP. Autophosphorylation produced a ≈ 20-fold increase in Src activity, consistent with earlier measurements (Fig. 5a). The auto-thiophosphorylated sample had approximately 10-fold higher activity than unphosphorylated Src, suggesting that thiophosphorylated Tyr416 is also able to stabilize the activated conformation (Fig. 5a). We confirmed these results by carrying out autothiophosphorylation reactions for varying lengths of time (0–60 min), then measuring kinase activity towards a synthetic peptide with the continuous assay. There was a thiophosphorylation-dependent increase in the initial rates of the peptide reactions that depended on the time of preincubation (Fig. 5b). The maximum change in rate was approximately 10-fold, after 60 min of thiophosphorylation.

**Fig. 6:**
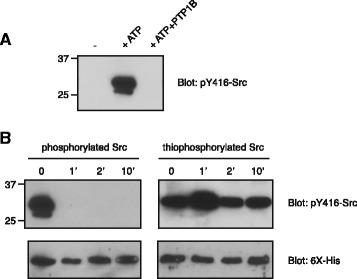
Thiophosphorylated Src is resistant to dephosphorylation. **a** Src catalytic domain was preincubated alone or in the presence of MgATP for 30 min at 30 °C. The samples were analyzed by SDS-PAGE, along with a sample of autophosphorylated Src that had been treated with immobilized GST-PTP1B phosphatase (30 min at 30 °C). Proteins in the gel were analyzed by Western blotting with anti-Src (pY416) antibody. **b** Phosphorylated or thiophosphorylated Src catalytic domain was treated with immobilized GST-PTP1B for the indicated amounts of time and analyzed by SDS-PAGE and Western blotting with anti-Src (pY416) antibody. The membrane was stripped of antibody and reprobed with anti-His tag antibody to confirm equal loading of the gel. This experiment was repeated three times with similar results

The metabolic stability of thiophosphorylated proteins (as compared to phosphorylated proteins) has been noted by several investigators. An early study showed that thiophosphorylated EGFR was dephosphorylated 20 to 40-fold more slowly than phosphorylated EGFR in A431 cell membranes [13]. Indeed, the use of thiophosphorylated lysozyme was a critical step in the purification of the first protein tyrosine phosphatases (PTPs) to be isolated [22]. Thiophosphorylated substrate analogs have been subsequently used as inhibitors of PTPs [23]. We investigated whether auto-thiophosphorylated Src is resistant to PTPs. First, we developed a Western blotting assay to conveniently measure thiophosphorylated Src. While a general anti-pTyr antibody did not specifically recognize thiophosphorylated Src, anti-phospho-Src (pY416) antibody showed a significant difference between unphosphorylated and thiophosphorylated Src (Additional file 3: Figure S3). The identity of the cellular PTP that dephosphorylates Tyr416 under physiological conditions is not certain, although various candidates have been proposed [24, 25]. For these experiments, we used purified PTP1B, a phosphatase that acts on Src under some conditions. We confirmed that PTP1B was able to dephosphorylate Tyr416 of Src under in vitro conditions (Fig. 6a). PTP1B rapidly dephosphorylated autophosphorylated Src, while auto-thiophosphorylated Src was resistant (Fig. 6b). Extended reactions with PTP1B (30–60 min) gave partial dephosphorylation, consistent with the degree of stabilization observed previously for EGFR (data not shown). Thus, auto-thiophosphorylated Src appears to be a stable, activated form of the kinase.

## Discussion

We report here that Src and other tyrosine kinases (including examples of receptor and nonreceptor tyrosine kinases) can catalyze auto-thiophosphorylation. The reaction is greatly enhanced in the presence of Ni^2+^. For Src, thiophosphorylation takes place primarily at Tyr416 within the activation loop, and produces a form of Src that is active and relatively resistant to the action of tyrosine phosphatases.

**Fig. 7:**
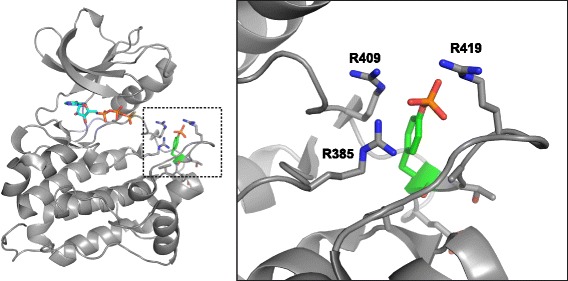
Structure of thiophosphorylated Tyr416. The *left panel* shows the crystal structure of Src in the presence of ATPγS (pdb code: 3DQW). ATPγS is shown in ball-and-stick format, and Tyr416 is shown in *green*. The *dotted box* is expanded in the right-hand panel. The (thio)phosphate group on Tyr416 is shown in orange, and the three Arg residues that coordinate the (thio)phosphate are shown in ball-and-stick format

There is crystallographic evidence for the ability of thiophosphorylated Tyr416 to stabilize the active conformation of Src. Azam et al. cocrystallized a mutant form of Src (T341I) with ATPγS [26]. (T341 is the so-called “gatekeeper” residue, a frequent site of resistance mutation in kinases targeted by small-molecule inhibitors). In the structure (pdb code: 3DQW), the adenine, ribose, and α-phosphate groups of ATPγS show well-defined electron density, with less well defined electron density for the β and γ phosphates. Tyr416 is phosphorylated, presumably by Src auto-thiophosphorylation, and makes interactions that are consistent with other activated Src structures (Fig. 7). In the crystal structure of activated Lck kinase (pdb code: 1QPC) [27], the activated conformation is stabilized by interactions between phosphorylated Tyr394 and arginines 387, 363, and 397; these Arg residues are conserved in Src (positions 409, 385, and 419, respectively). The activation loop is well ordered, and resembles the activated conformation seen in other Src kinase structures.

The stoichiometry of thiophosphorylation after 30 min was 0.91 mol/mol (Fig. 3b). The structure of thiophosphorylated Src suggests that this modification should increase enzymatic activity, and we have confirmed that this is the case. The activity of auto-thiophosphorylated Src is significantly higher than that of unphosphorylated Src, as measured toward a synthetic peptide substrate (Fig. 5). Thiophosphorylated Src had activity that was roughly one half that of phosphorylated Src (Fig. 5a). This may reflect subtle differences in the conformation of phosphorylated vs. thiophosphorylated Tyr416 that result in changes in catalytic efficiency.

Thiophosphorylated Src was resistant to dephosphorylation by PTP1B tyrosine phosphatase (Fig. 6). This is consistent with earlier reports on a wide variety of tyrosine phosphatases; PTPs are able to bind peptides and proteins containing thiophosphorylated tyrosine, but the catalytic rates of dephosphorylation are sluggish [13, 22, 23]. Thiophosphotyrosyl analogs of substrates bind to the active sites of PTPs, and act as competitive inhibitors. Acidic residues, such as those found N-terminal to Tyr416 of Src (sequence: Glu-Asp-Asn-Glu-Tyr) are often important specificity determinants for binding to PTPs [23, 28].

We and others have previously observed that the inclusion of thiophilic divalent cations such as Co^2+^ or Ni^2+^ enhances the thiophosphorylation activity of protein kinases [10, 16]. Inclusion of Mn^2+^ together with Mg^2+^ resulted in high levels of Abl thiophosphorylation, even in the presence of micromolar concentrations of ATP, a development that could allow the study of thiophosphorylation in cell extracts [15]. Previous studies of tyrosine kinases focused on the ability of kinases to thiophosphorylate exogenous substrates. There is one previous study of the functional consequences of auto-thiophosphorylation by a eukaryotic protein kinase. The Ser/Thr kinase calmodulin-dependent protein kinase II (CaM-kinase II) is thiophosphorylated at Thr286 and Thr287 upon reaction with ATPγS [12]. The kinetic properties of thiophosphorylated CaM-kinase II were found to be similar to those of the phosphorylated enzyme. The stability of the thiophosphate linkage allowed the investigators to show that autophosphorylation is required for full enzyme activation [14]. In a similar manner, thiophosphorylated derivatives could serve as stable, persistently-activated mimics of tyrosine kinases.

## Conclusions

We show that: (1) In the presence of Ni^2+^, Src and other tyrosine kinases catalyze auto-thiophosphorylation using ATPγS as a phosphodonor; (2) Auto-thiophosphorylation of Src occurs predominantly at Tyr416 in the activation loop; (3) Src auto-thiophosphorylation increases the enzyme’s catalytic activity; (4) Tyr416-thiophosphorylated Src is resistant to dephosphorylation by PTP1B phosphatase, and could serve as a stable, persistently-activated mimic of Src.

## Methods

### Materials

The catalytic domains of Src and Hck were expressed in bacteria and purified as previously described by Seeliger, Kuriyan, and colleagues [21]. The catalytic domains of Ack1 and IGF1R kinases were expressed in Sf9 cells using recombinant baculoviruses, as previously described [29, 30]. ATP, adenosine 5′-(3-thiotriphosphate) (ATPγS), and PK/LDH were purchased from Sigma. The anti-Src (pY419; equivalent to chicken c-Src pY416) antibody was from Biosource, and anti-pTyr antibody (4G10) was from Millipore. [^35^S]-labeled ATPγS was from Perkin-Elmer. Dithiothreitol (DTT), acetonitrile (ACN), ammonium bicarbonate, trifluoroacetic acid (TFA), and iodoacetamide (IAA) were from Thermo Fisher Scientific (Waltham, MA). Trypsin Gold, mass spectrometry grade, was from Promega (Madison, WI). Tris-HCl (10 %) non-denaturing gels were purchased from Bio-Rad.

### Kinase assays

Two kinase assays were employed : (1) Continuous kinase assays were performed by a coupled spectrophotometric assay [6]. In this assay, the production of ADP is coupled to the oxidation of NADH measured as a reduction in absorbance at 340 nm. All experiments were carried out at 30 °C. Reactions were performed in buffer containing 100 mM Tris pH 7.5, 1 mM phosphoenolpyruvate, 0.28 mM NADH, 89 units/ml pyruvate kinase and 124 units/ml lactate dehydrogenase, with varying concentrations of enzyme and divalent cations. In some experiments, a peptide substrate (AEEEIYGEFEAKKKKG) was included. (2) Peptide phosphorylation was also measured using [γ-^32^P]-ATP and a phosphocellulose paper binding assay [31]. Reactions were performed in 20 mM Tris-HCl (pH 7.4), 10 mM MgCl_2_, 0.25 mM ATP, varying concentrations of peptide substrate, and [γ-^32^P] -ATP (100–500 cpm/pmol).

### Mass spectrometry

Src kinase (3.2 μM) was incubated with 2 mM ATPγS and 3 mM NiCl_2_ for 1 h, 45 min at 30°. A control sample was prepared by carrying out a similar reaction without ATPγS. Both samples were analyzed by SDS-PAGE. Gel bands were cut out, destained and digested with trypsin essentially as described [32] with minor modifications. The gel bands were not reduced and alkylated as iodoacetamide also reacted with the thiol group in the thiophosphate and produced a more complicated fragmentation pattern in the mass spectrometer.

The dried peptide mix was reconstituted in a solution of 2 % acetonitrile (ACN), 2 % formic acid (FA) for MS analysis. Peptides were loaded with the autosampler directly onto a 2 cm C18 PepMap pre-column (Thermo Scientific, San Jose, CA) which was attached to a 50 cm EASY-Spray C18 column (Thermo Scientific). Peptides were eluted from the column using a Dionex Ultimate 3000 Nano LC system with a 10 min gradient from 2 % buffer B to 35 % buffer B (100 % acetonitrile, 0.1 % formic acid). The gradient was switched from 35 to 85 % buffer B over 1 min and held constant for 2 min. Finally, the gradient was changed from 85 % buffer B to 98 % buffer A (100 % water, 0.1 % formic acid) over 1 min, and then held constant at 98 % buffer A for 5 more minutes. The application of a 2.0 kV distal voltage electrosprayed the eluting peptides directly into the Thermo Fusion Tribrid mass spectrometer equipped with an EASY-Spray source (Thermo Scientific). Initial experiments were performed to identify potential phosphorylation and thiophosphorylation sites by running each digest separately with a data dependent method to acquire as many MS/MS in a 3 s span. This data was analyzed for the presence of phosphorylation and thiophosphorylation and subsequently the samples were rerun in which the mass spectrometer was set to a targeted analysis method to only acquire CID MS/MS of the expected unphosphorylated (m/z 612.29 and 617.81, for peptide LIEDNEYTAR and WTAPEAALYGR, respectively) and phosphorylated (m/z 652.28 and 657.79) and thiophosphorylated (m/z 660.27 and 665.79) peptides. These MS/MS scans were acquired in the Orbitrap at 15,000 resolution with a scan range of m/z 200–1300 and 200–1200, respectively. Mass spectrometer-scanning functions and HPLC gradients were controlled by the Xcalibur data system (Thermo Scientific). The acquired MS data were analyzed manually to confirm the precursor mass, fragmentation ions, and phosphorylations and thiophosphorylations in the targeted peptides.

### Western blotting

Reactions were separated on 10 % SDS-PAGE, and transferred onto a polyvinylidene difluoride membrane. Proteins were detected by Western blotting with antiphosphotyrosine and anti-pY419 antibodies.

### Native gel analysis

Src kinase (8.5 μM) was incubated in 20 mM Tris-HCl pH 7.5 at 30 °C with either (1) 5 mM ATPγS, 10 mM NiCl_2_ or (2) 5 mM ATP, 10 mM MgCl_2_. Reactions were stopped at various time points by addition of 100 mM EDTA and analyzed by native PAGE using 10 % Tris–HCl gels. The thiophosphorylated and phosphorylated forms of Src were visualized by staining with Coomassie blue.

### Stoichiometry measurement

Reactions (30 °C) contained Src kinase (1 μM), 30 mM Tris-HCl (pH 7.5), 10 mM NiCl_2_, and [^35^S]-labeled ATPγS (30 pmol). Aliquots of the reactions were withdrawn at various time points, and ^35^S-labeled Src was spotted onto Whatman 3MM paper, washed with 5 % trichloroacetic acid at 55 °C, and counted by liquid scintillation counting [7].

## Abbreviations

Ack1, activated Cdc42-associated kinase; ACN, acetonitrile; ATPγS, adenosine 5′-O-(3-thio)triphosphate; CaM, calcium/calmodulin; DTT, dithiothreitol; EDTA, ethylenediaminetetraacetic acid; EGFR, epidermal growth factor receptor; FA, formic acid; Hck, hematopoietic cell kinase; HPLC, high pressure liquid chromatography; IAA, iodoacetamide; IGF1R, insulin-like growth factor 1 receptor; MS, mass spectrometry; NADH, nicotinamide adenine dinucleotide; PK/LDH, pyruvate kinase/lactate dehydrogenase; PKA, protein kinase A; PTP1B, protein tyrosine phosphatase 1B; TFA, trifluoroacetic acid

## Additional files

Additional file 1: Figure S1. Details of the MS/MS fragmentations for the two major thiophosphorylated peptides from Src. (A) LIEDNEY^416^(thiophos)TAR; (B) WTAPEAALY^436^(thiophos)GR. (PDF 226 kb) Additional file 2: Figure S2. LC profiles for the two major thiophosphorylated peptides from Src. The ion current signals for the unmodified and thiophosphorylated peptides are shown next to the chromatographic peaks. (A) LIEDNEY^416^TAR; (B) WTAPEAALY^436^GR. (PDF 363 kb) Additional file 3: Figure S3. Western blotting method to detect thiophosphorylated Src. Src (catalytic domain, 295 nM) was incubated alone (unphos), with 1 mM ATP and 5 mM MgCl_2_ (phos), or with 1 mM ATPγS and 10 mM NiCl_2_ (thiophos) for 30 min at 30 °C. The reactions were analyzed by SDS-PAGE and Western blotting with anti-pTyr antibody (top) and with anti-Src (pY416) antibody (bottom). In the right-hand lane of the gel, four times the amount of Src was loaded as in the other lanes. (PDF 466 kb)
