# Supplementary material for: Auto-thiophosphorylation activity of Src tyrosine kinase
Source: BMC Biochem. 2016 Jul 7;17:13. doi: 10.1186/s12858-016-0071-z (PMC4936181; doi:10.1186/s12858-016-0071-z)
Supplement: Additional file 1: Figure S1. — Details of the MS/MS fragmentations for the two major thiophosphorylated peptides from Src. (A) LIEDNEY416(thiophos)TAR; (B) WTAPEAALY436(thiophos)GR. (PDF 226 kb) [file 12858_2016_71_MOESM1_ESM.pdf]

**A**

| b | | | | y | y+2 |
|---|---|---|---|---|---|
| --- | 1 | L | 10 | --- | --- |
| 227.1754 | 2 | I | 9 | 1206.4492 | 603.7282 |
| 356.2180 | 3 | E | 8 | 1093.3651 | 547.1862 |
| 471.2449 | 4 | D | 7 | 964.3225 | 482.6649 |
| 585.2879 | 5 | N | 6 | 849.2956 | 425.1514 |
| 714.3305 | 6 | E | 5 | 735.2527 | 368.1300 |
| 973.3368 | 7 | Y(95.943) | 4 | 606.2101 | 303.6087 |
| 1074.3845 | 8 | T | 3 | 347.2037 | 174.1055 |
| 1145.4216 | 9 | A | 2 | 246.1561 | 123.5817 |
| --- | 10 | R | 1 | 175.1190 | 88.0631 |

**B**

| b | | | | y | y+2 |
|---|---|---|---|---|---|
| --- | 1 | W | 11 | --- | --- |
| 288.1343 | 2 | T | 10 | 1144.4852 | 572.7462 |
| 359.1714 | 3 | A | 9 | 1043.4375 | 522.2224 |
| 456.2241 | 4 | P | 8 | 972.4004 | 486.7038 |
| 585.2267 | 5 | E | 7 | 875.3476 | 438.1775 |
| 656.3039 | 6 | A | 6 | 746.3050 | 373.6562 |
| 727.3410 | 7 | A | 5 | 675.2679 | 338.1376 |
| 840.4250 | 8 | L | 4 | 604.2308 | 302.6190 |
| 1099.4314 | 9 | Y(95.943) | 3 | 491.1467 | 246.0770 |
| 1156.4528 | 10 | G | 2 | 232.1404 | 116.5738 |
| --- | 11 | R | 1 | 175.1190 | 88.0631 |
