# Supplementary figures and images for: Auto-thiophosphorylation activity of Src tyrosine kinase
Source: BMC Biochem. 2016 Jul 7;17:13. doi: 10.1186/s12858-016-0071-z (PMC4936181; doi:10.1186/s12858-016-0071-z)

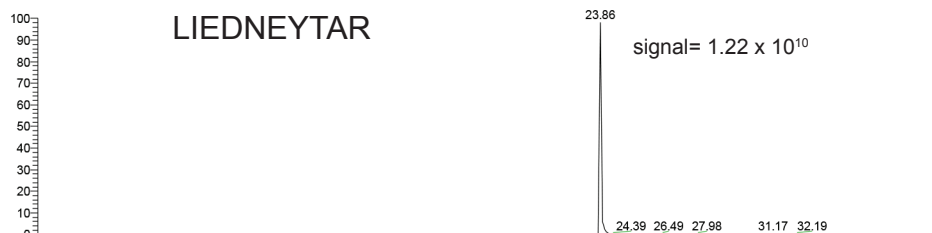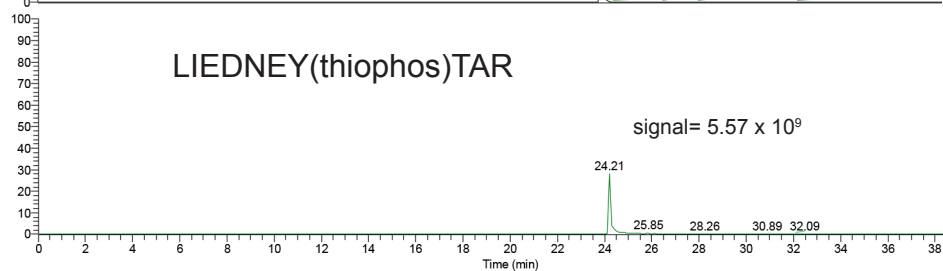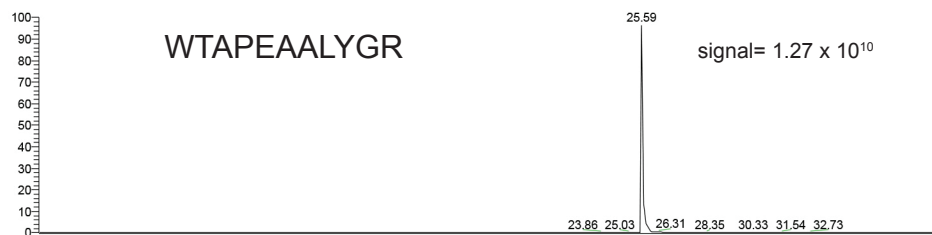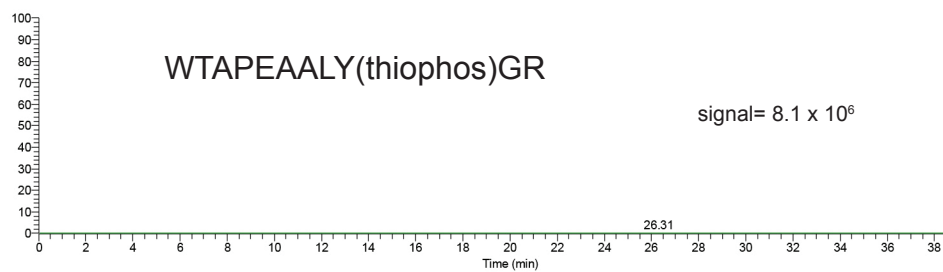

Supplement: Additional file 2: Figure S2. — LC profiles for the two major thiophosphorylated peptides from Src. The ion current signals for the unmodified and thiophosphorylated peptides are shown next to the chromatographic peaks. (A) LIEDNEY416TAR; (B) WTAPEAALY436GR. (PDF 363 kb) [file 12858_2016_71_MOESM2_ESM.pdf]

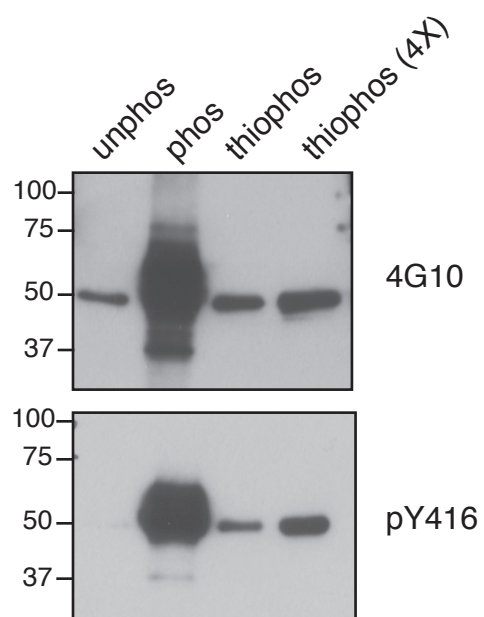

Supplement: Additional file 3: Figure S3. — Western blotting method to detect thiophosphorylated Src. Src (catalytic domain, 295 nM) was incubated alone (unphos), with 1 mM ATP and 5 mM MgCl2 (phos), or with 1 mM ATPγS and 10 mM NiCl2 (thiophos) for 30 min at 30 °C. The reactions were analyzed by SDS-PAGE and Western blotting with anti-pTyr antibody (top) and with anti-Src (pY416) antibody (bottom). In the right-hand lane of the gel, four times the amount of Src was loaded as in the other lanes. (PDF 466 kb) [file 12858_2016_71_MOESM3_ESM.pdf]
